# Computer modeling of the peculiarities in the interaction of IL-1 with its receptors in schizophrenia

**DOI:** 10.18699/vjgb-24-38

**Published:** 2024-06

**Authors:** N.Yu. Chasovskikh, A.A. Bobrysheva, E.E. Chizhik

**Affiliations:** Siberian State Medical University of the Ministry of Healthcare of the Russian Federation, Tomsk, Russia; Siberian State Medical University of the Ministry of Healthcare of the Russian Federation, Tomsk, Russia; Siberian State Medical University of the Ministry of Healthcare of the Russian Federation, Tomsk, Russia

**Keywords:** IL-1, schizophrenia, molecular modeling, SNP, single-nucleotide polymorphisms, PRISM, IL-1, шизофрения, моделирование, SNP, однонуклеотидные полиморфизмы, PRISM

## Abstract

One of the primary theories regarding the development of schizophrenia revolves around genetics, indicating the involvement of hereditary factors in various processes, including inflammation. Research has demonstrated that inflammatory reactions occurring in microglia can impact the progression of the disease. It has also been established that genetically determined changes in IL-1 can contribute to schizophrenia, thereby confirming the role of the IL-1 gene cluster in disease susceptibility. The aim of this study is a computer-based assessment of the structural interactions of IL-1 proteins with their receptors in schizophrenia. The study utilized the DisGeNET database, enabling the assessment of the reliability of identified IL-1 polymorphisms. Polymorphisms were also sought using NCBI PubMed. The NCBI Protein service was employed to search for and analyze the position of the identified polymorphisms on the chromosome. Structures for modeling were extracted from the Protein Data Bank database. Protein modeling was conducted using the SWISS-MODEL server, and protein interaction modeling was performed using PRISM. Notably, this study represents the first prediction of the interactions of IL-1α, IL-1β, and IL- 1RA proteins, taking into account the presence of single-nucleotide polymorphisms associated with schizophrenia in the sequence of the corresponding genes. The results indicate that the presence of SNP rs315952 in the IL-1RA protein gene, associated with schizophrenia, may lead to a weakening of the IL-1RA binding to receptors, potentially triggering the initiation of the IL-1 signaling pathway by disrupting or weakening the IL-1RA binding to receptors and facilitating the binding of IL-1 to them. Such alterations could potentially lead to a change in the immune response. The data obtained contribute theoretically to the development of ideas about the molecular mechanisms through which hereditary factors in schizophrenia influence the interactions of proteins of the IL-1 family, which play an important role in the processes of the immune system.

## Abstract (ru)

Одной из основных теорий развития шизофрении является генетическая, свидетельствующая о вовлечении наследственных факторов в различные процессы, в том числе воспаление. Показано, что воспалительные реакции, протекающие в микроглии, могут влиять на развитие заболевания. Также установлено, что генетически обусловленные изменения IL-1 могут способствовать шизофрении, подтверждая роль кластера генов IL-1 в восприимчивости к болезням. Целью работы была компьютерная оценка структурных взаимодействий белков IL-1 с их рецепторами при шизофрении. Использовалась база данных DisGeNET, позволяющая оценить достоверность выявленных полиморфизмов IL-1. Проведен поиск полиморфизмов с помощью NCBI PubMed. Сервис NCBI Protein использовался для поиска и анализа положения на хромосоме найденных полиморфизмов. Из базы данных Protein Data Bank были извлечены структуры для проведения моделирования. Моделирование белков выполнялось с помощью сервера SWISS-MODEL, а моделирование белковых взаимодействий – с помощью PRISM. В настоящем исследовании впервые проведено прогнозирование взаимодействий белков IL-1α, IL-1β и IL-1RA с учетом наличия в последовательности соответствующих генов однонуклеотидных полиморфизмов, ассоциированных с шизофренией. Показано, что наличие ассоциированного с шизофренией полиморфизма rs315952 гена белка IL-1RA может привести к ослаблению связи IL-1RA с рецепторами и, предположительно, к запуску сигнального пути IL-1 путем разрыва либо ослабления связи IL-1RA с рецепторами и связыванием IL-1 с ними, что, возможно, вызовет изменение иммунного ответа. Полученные данные вносят теоретический вклад в развитие представлений о молекулярных механизмах влияния наследственных факторов шизофрении на взаимодействия белков семейства IL-1, играющих важную роль в процессах иммунной системы

## Introduction

The investigation of the causes of multifactorial diseases, characterized by complex inheritance and associated with the action of multiple genes (Bochkov, 2011), is a current challenge in contemporary medical biological science. When studying such diseases, special attention is given to their potential associations with single nucleotide polymorphisms (SNPs), as well as the involvement of the corresponding genes in the implementation of molecular mechanisms underlying pathologies

Currently, a pressing issue is the exploration of the mechanisms underlying the development of such a prevalent disorder as schizophrenia. This condition has several etiopathogenetic concepts, with one of the main theories being genetic. It suggests the involvement of genetic factors in various physiological processes of the body, including inflammatory processes. The activation of the inflammatory response system, associated with the pathophysiology of schizophrenia, has been demonstrated in numerous studies (Xu, He, 2010; Sommer et al., 2015; Kapelski et al., 2016; Miyaoka et al., 2017; Müller, 2019). Studies on animal models of schizophrenia, including mice and primates, indicate that inflammatory reactions during pregnancy may influence brain development and contribute to the etiology of this disorder (Frodl, Amico, 2014). It has been shown that microglial cells are activated in schizophrenia and play a crucial role in inflammatory processes (Müller, 2019). Additionally, the nonsteroidal anti-inflammatory drug Celecoxib has been found to exert therapeutic effects on patients with schizophrenia. Considering these findings, immunomodulation is currently widely discussed as a potential approach to the treatment of this disorder (Müller, 2019).

Clinical case descriptions of patients undergoing bone marrow transplantation demonstrate the inflammatory nature of schizophrenia. For instance, T. Miyaoka et al. (2017) presented the case of a 24-year-old man with treatment-resistant schizophrenia who underwent bone marrow transplantation for acute myeloid leukemia. After the procedure, he showed a significant reduction in schizophrenia symptoms without the use of neuroleptics. I.E. Sommer et al. (2015) described a reverse case where schizophrenia was transmitted from a sibling. At present, the mechanism of changes introduced by bone marrow transplantation from a healthy individual influencing the development of schizophrenia is not fully understood. However, it has been shown that this process normalizes microglial changes, which are significant for this disorder (Miyaoka et al., 2017). While the examination of individual cases cannot definitively confirm the immune pathogenesis of schizophrenia, the involvement of the immune system may be one of the key factors in the development of this disorder (Sommer et al., 2015)

It has been demonstrated that genetically determined changes in the regulation of IL-1 metabolism, one of the key components of the immune response, may contribute to schizophrenia, thereby supporting the role of the IL-1 gene cluster in disease susceptibility (Zanardini et al., 2003). Pro-inflammatory cytokines can modify neurotransmitter metabolism, influencing the development of the nervous system. IL-1 participates in both acute and chronic neurodegeneration, suggesting that cytokines induced by the activation of the IL-1 signaling pathway may play a pivotal role both in the acute phase of the disease and during developmental stages of the brain that affect an individual’s susceptibility to schizophrenia- related factors in later life (Katila et al., 1999).

Accumulated data to date provide an opportunity for a more detailed examination of the influence of individual cytokine genes, particularly IL-1, on the mechanisms underlying schizophrenia development. Bioinformatic methods enable the exploration of changes in gene sequences associated with this disorder and an assessment of the properties of the corresponding protein molecules. This includes their involvement in interleukin receptor interactions, impacting the realization of the pro-inflammatory effects of IL-1. This will expand theoretical knowledge and identify approaches for further investigations into potential mechanisms of the immune system’s involvement in schizophrenia development

The objective of this study is a computer-based assessment of the interactions between IL-1 proteins and their receptors in the context of schizophrenia

## Materials and methods

We investigated the genetic factors associated with schizophrenia using the DisGeNET platform renowned for hosting one of the largest publicly available collections of genes and variants linked to human diseases (Piñero et al., 2020). The search for SNPs and proteins related to the IL-1 family was conducted through the NCBI (National Center for Biotechnology Information) PubMed service and the Protein database (Sayers et al., 2021).

To ensure the reliability of the data obtained from the DisGeNET platform, we assessed the identified polymorphisms using the Evidence Index. An Evidence Index (EI) of 1 signifies unanimous support for Gene-Disease Associations (GDA) or Variant-Disease Associations (VDA) across all publications. A value of EI < 1 indicates the absence of a correlation between the gene/variants and the disease (Piñero et al., 2020).

Following the selection of polymorphisms in genes encoding proteins associated with the IL-1 family, we analyzed their chromosomal positions using the NCBI resource functionality (Sherry et al., 2001). It was imperative to locate the polymorphisms within the coding region for modeling the corresponding proteins

The amino acid sequences for protein modeling were sourced from the NCBI Protein database (Sayers et al., 2021). Subsequently, we manually replaced the amino acids in the sequences based on the positions of the polymorphisms. Protein modeling using the obtained sequences was carried out using the SWISS-MODEL protein structure modeling server (Waterhouse et al., 2018).

We extracted the IL-1R1+IL-1RAP+IL-1β complex from the Protein Data Bank (PDB) database, which houses known spatial structures of proteins. Subsequently, IL-1β was removed from this structure as our analysis focused on the receptor complex without interleukin

The receptor complexes obtained were imported into Protein Interactions by Structural Matching (PRISM) (Baspinar et al., 2014), where their surfaces underwent structural comparison with template interfaces – previously identified binding regions. An interface (binding region) is defined as a pair of sets of amino acid residues {(A1, ..., AN), (B1, ..., BM)}, where for any amino acid residue Ai from protein A, there is at least one amino acid residue Bi from protein B. This occurs in such a way that the distance between these residues does not exceed a specified threshold (typically ranging from 6 to 12 Å) (Hadarovich et al., 2020). Within the binding region, hot spots exist – amino acid residues contributing significantly to binding energy (Tuncbag et al., 2012).

PRISM operates as an algorithm for predicting and modeling protein interactions through structural matching, encompassing four key stages: extraction of the target protein surface; assessment of structural similarity with template interface partners; superimposition of protein surface areas resembling the template interface on the template; flexible refinement of the obtained complexes, and energy calculation (Aytuna et al., 2005; Tuncbag et al., 2011).

Through the modeling of molecular interactions, the PRISM service provides an interface for forecasting, the complex structure, and an energy indicator. The latter signifies binding energy, denoting the minimum work required to separate the system into its constituent particles. It characterizes system stability and consistently carries a negative value, with the system boasting the lowest binding energy considered the most stable (Acuner Ozbabacan et al., 2014).

An energy threshold value of –10 kJ/mol was employed to identify energetically favorable predictions. Interactions demonstrating conformational advantage, backed by experimental data and IS-assessment (interface similarity assessment), with an output energy less than –10 kJ/mol were deemed favorable (Gao, Skolnick, 2011; Kuzu et al., 2013). The IS score, a metric for evaluating protein-protein interaction predictions, takes into account not only geometric distances but also the preservation of interfacial contacts. For the PRISM algorithm, an IS score greater than 0.12 is considered acceptable (Gao, Skolnick, 2011).

To visualize the localization of amino acid substitutions and interactions of IL-1 with receptors in the obtained protein complexes, the YASARA program (Krieger, Vriend, 2014) was utilized.

## Results

Identification of molecules initiating the IL-1 signaling pathway

**Fig. 1.**
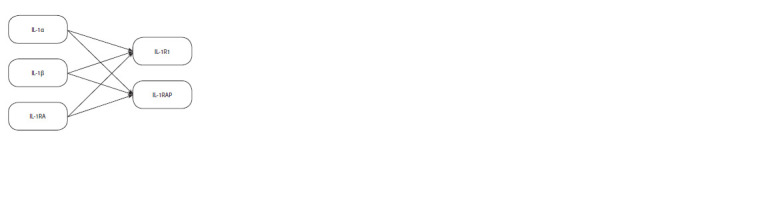
IL-1 molecules and their interaction with cell receptors (based on Acuner Ozbabacan et al., 2014; Dinarello, 2018).

The structures of IL-1α, IL-1β, and IL-1RA proteins, crucial for initiating the IL-1 pathway, underwent examination (Dinarello, 1994). These proteins interact with specific receptors IL-1R1 and IL-1RAP (Acuner Ozbabacan et al., 2014). Subsequently, we evaluated the presence of polymorphisms in the genes of IL-1α, IL-1β, IL-1RA, IL-1R1, and IL-1RAP proteins, modeling their interactions according to the scheme presented in Figure 1.

Search for SNPs in IL-1 genes associated with schizophrenia

A search in the DisGeNET catalog identified four singlenucleotide polymorphisms in genes initiating the IL-1 pathway associated with schizophrenia

For IL-1α, SNPs rs113129609 and rs1800587 were found. While the EI for rs113129609 was 1, the corresponding article did not confirm its presence. For rs1800587, with an EI index less than 0.001, evidence was lacking. The rs16944 polymorphism in IL-1β, with an EI of 1, was supported by several studies (Shirts et al., 2006; Xu, He, 2010; Fatjó-Vilas et al., 2012), and the polymorphism was included in the list for further investigation. The rs1794068 polymorphism for IL-1RA had an EI less than 0.001, and further investigation was not pursued.

A PubMed search yielded 39 articles, and polymorphisms were extracted and listed in Table 1.

**Table 1.**
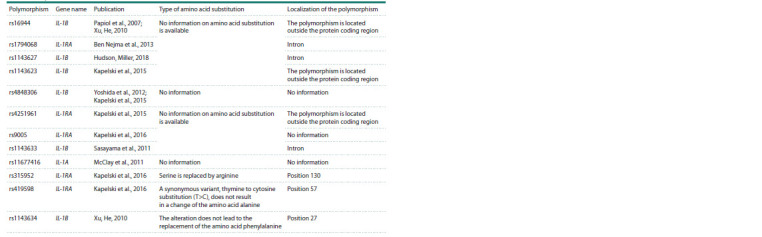
Analysis of the localization and substitution of an amino acid in the sequence

Analysis of the localization of SNPs of genes initiating the IL-1 signaling pathway

Localization and information on amino acid substitution for each polymorphism were analyzed using the dbSNP resource (Sherry et al., 2001) (Table 1). The IL-1RA rs315952 polymorphism, involving the substitution of serine with arginine, was identified for further modeling

Modeling of proteins initiating the IL-1 signaling pathway

Since the rs315952 polymorphism is located in the IL-1RA amino acid sequence, it was selected for modeling. The original sequence was extracted from the NCBI Protein sequence database: >NP_776214.1 interleukin-1 receptor antagonist protein isoform 1 precursor [Homo sapiens].

Three-dimensional structures of IL-1RA were modeled with and without the polymorphism using the SWISS-MODEL service. The obtained molecular models were saved as “.pdb” files.

Since IL-1RA interacts with IL-1R1, IL-1RAP, and the IL- 1R1+IL-1RAP complex (Fig. 1), three-dimensional structures of the corresponding proteins were required for modeling and analysis. The PDB structure of the IL-1β signaling complex was obtained, including IL-1β (chains A, D), IL-1R1 (chains B, E), and IL-1RAP (chains C, F). The IL-1R1+IL- 1RAP complex, IL-1RAP, and IL-1R1 were obtained using the PyMol program.

**Fig. 2.**
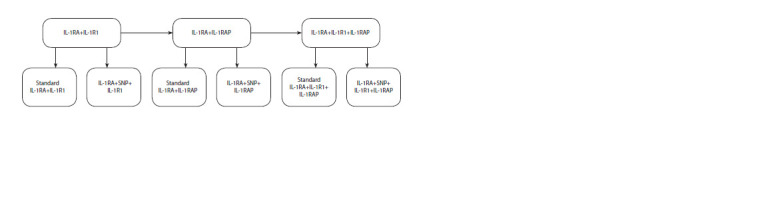
Stages of protein interactions modeling.

Modeling protein interactions initiating the IL-1 pathway Modeling of interactions followed the scheme presented in Figure 2. Interactions of standard (non-polymorphic) IL- 1RA with IL-1R1, IL-1RAP, and the IL-1R1+IL-1RAP receptor complex were modeled and analyzed sequentially. Subsequently, interactions with IL-1RA rs315952 were also modeled.

Modeling IL-1RA interactions with IL-1R1

Modeling interaction of standard IL-1RA with IL-1R1. In the obtained models, the minimum energy indicators demonstrated interaction according to the 1itbAB template, characterizing the most probable interaction where the structure is maximally stable (Table 2).

**Table 2.**
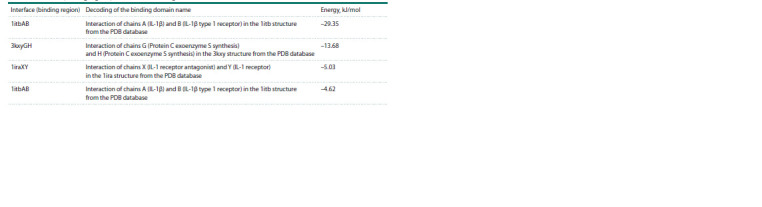
Interfaces (binding regions) and interaction energies of standard IL-1RA with IL-1R1

Considering the rs315952 polymorphism (Table 1) involving the replacement of serine by arginine at position 130, the interaction at this point was evaluated under normal and polymorphic conditions. According to the contact list of template residues, serine at position 130 of the IL-1RA molecule binds to leucine at position 237 of IL-1R1 (see Table 4).

**Table 4.**
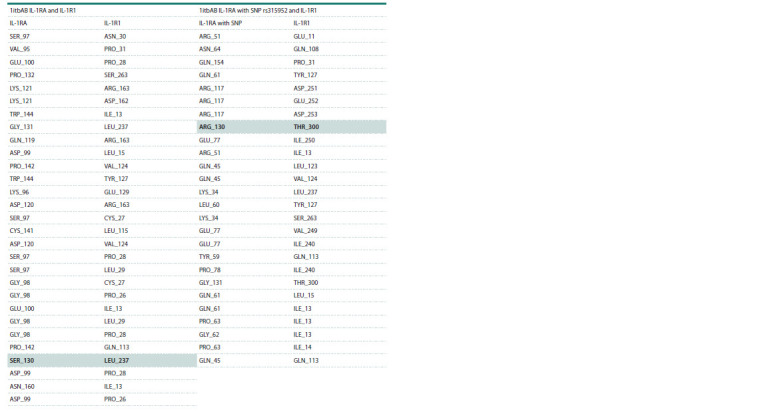
Contacts of interface residues of 1itbAB IL-1RA and IL-1R1 and 1itbAB IL-1RA with SNP rs315952 and IL-1R1

Modeling interaction of IL-1RA with the rs315952 polymorphism with IL-1R1. The structures of IL-1RA molecules with the rs315952 polymorphism and IL-1R1 were utilized for modeling

In the results of modeling this interface, minimum energy values were found for the 1iraXY template (Table 3). However, as the interaction without the polymorphism in the IL-1RA structure (IL-1RA+IL-1R1) showed minimal interaction energy according to the 1itbAB template, the energy should be compared using the same template.

**Table 3.**
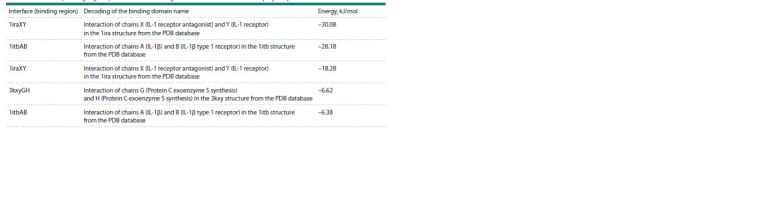
Interfaces (binding regions) and interaction energies of IL-1RA with the rs315952 polymorphism with IL-1R1

Comparison between Tables 2 and 3 suggests that the interaction of IL-1RA with the rs315952 polymorphism with IL- 1R1 (–30.08 kJ/mol) is the most energetically advantageous. However, when comparing energies using the same 1itbAB template, this interaction becomes less energetically favorable (–29.35 kJ/mol and –28.18 kJ/mol, respectively). This suggests that in the presence of the rs315952 polymorphism in IL1-RA (serine substitution at position 130 for arginine (Table 4)), the interleukin-receptor interaction complex weakens, becoming less stable and more susceptible to decay.

Thus, based on the interactions of IL-1RA with the rs315952 polymorphism with IL-1R1, we cannot draw a definitive conclusion regarding the polymorphism’s impact on its involvement in initiating the IL-1 signaling pathway. However, the modeled interactions indicate that the polymorphism participates in the formation of the protein-protein complex

Modeling interactions of IL-1RA with IL-1RAP

Two investigations were conducted for modeling interactions: the interaction of standard IL-1RA with IL-1RAP and IL-1RA with the rs315952 polymorphism with IL-1RAP. In both cases, the algorithm did not create a model of protein interaction

Modeling interactions of IL-1RA with the IL-1R1+IL-1RAP complex

Modeling interaction of standard IL-1RA with the IL- 1R1+IL-1RAP complex. The interaction of IL-1RA with the receptor complex using the 1iraXY template showed a stable interaction (–34.27 kJ/mol) (Table 5). However, analyzing the interaction using the 1itbAB template revealed a very weak interaction (–2.67 kJ/mol).

**Table 5.**
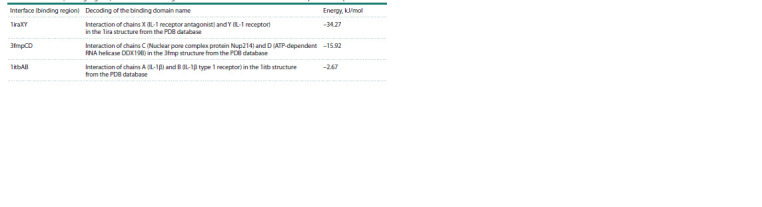
Interfaces (binding regions) and interaction energies of standard IL-1RA with the IL-1R1+IL-1RAP protein complex

**Table 6.**
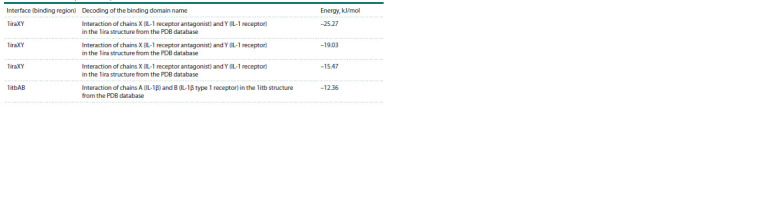
Interface (binding region) and interaction energies of IL-1RA with the rs315952 polymorphism with the IL-1R1+IL-1RAP protein complex

According to the contact list of template residues, serine at position 130 is also a hotspot (Table 7).

**Table 7.**
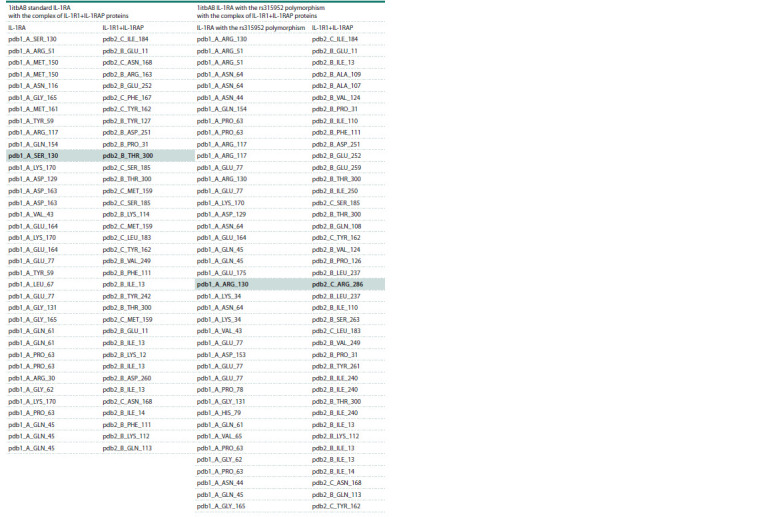
Contacts of residues in the interface of 1itbAB standard IL-1RA with the complex of IL-1R1+IL-1RAP proteins and 1itbAB IL-1RA with the rs315952 polymorphism with the complex of IL-1R1+IL-1RAP proteins

The results in Table 5 indicate that the 1itbAB template is suitable for interaction with the added IL-1RAP protein, but its stability is almost minimal, implying the formed complex will quickly break down. Therefore, for further interaction analysis, we use the 1iraXY template.

Modeling interaction of IL-1RA with the rs315952 polymorphism with the IL-1R1+IL-1RAP complex. According to the contact list of template residues, arginine is also a hotspot. The simulation results presented in Table 6 show that the minimum energy of the complex is observed with the 1iraXY template at –25.27 kJ/mol.

Comparing interactions with the complex without polymorphism using the 1iraXY template, it is evident that the minimum energy without polymorphism is –34.27 kJ/mol, while with polymorphism it is –25.27 kJ/mol. Thus, it can be hypothesized that the studied rs315952 polymorphism affects the formation of IL-1RA binding with the IL-1R1+IL-1RAP receptor complex, creating a less stable complex more prone to decay

The study results allow us to make an assumption that the p.Ser130Arg mutation in the IL-1RA protein gene may lead to the formation of a weakened complex between IL-1RA and the associated receptors IL-1R1+IL-1RAP, which could impact schizophrenia mechanisms

## Discussion

The functions of IL-1 family molecules are primarily associated with innate immunity. While inflammation normally acts as a protective mechanism, it can cause damage to the body when it becomes uncontrollable (Dinarello, 2018). IL-1 has been implicated in neuronal cell damage (Allan et al., 2005), and excessive phagocytosis may contribute to pathologies in Alzheimer’s disease, schizophrenia, and aging (Vilalta, Brown, 2018). IL-1 triggers phagocytosis in the brain by acting as a chemoattractant for neutrophils. Initiating the IL-1 signaling pathway also leads to the release of cytokines TNFα and IFN-γ, which activate macrophages (Sasayama et al., 2011).

Studies confirm an increase in the level of IL-1 in the blood of individuals with schizophrenia (Chu et al., 2018; Zhou et al., 2019). The reporter system of genetic knockout in mice, used to track the reciprocal deletion or expression of the IL-1 receptor (IL-1R1) in endothelial cells, ventricles, peripheral myeloid cells, microglia, astrocytes, and neurons, revealed that endothelial IL-1R1 is necessary and sufficient for mediating pain behavior. It is also shown to stimulate the proliferation of leukocytes in the central nervous system (CNS) and attenuate neurogenesis. Ventricular IL-1R1 is critical for the proliferation of monocytes in the CNS. Although microglia does not express IL-1R1, stimulation of endothelial cells with IL-1 leads to the induction of IL-1 in microglia (Liu et al., 2019).

The IL-1RA protein, which is an antagonist of IL-1 receptors and has an anti-inflammatory function (Dinarello, 1994), has also been found to be associated with schizophrenia (Kim et al., 2004). Preliminary results suggest that the IL-1RA protein gene may contribute to the ventricular changes observed in patients with this disease (Papiol et al., 2005).

An association has been found between single nucleotide polymorphisms in proteins involved in the IL-1 pathway and the risk of developing schizophrenia (Xu, He, 2010). There is a tendency for the association of the GAGG haplotype (rs1143627, rs16944, rs1143623, rs4848306) of the ILB gene; TG haplotypes (rs315952, rs9005) and TT61 rs5254 (rs4) of IL1RN, and CT haplotype (rs4251961, rs419598) in IL1RN with the risk of schizophrenia. Statistically significant association is shown for rs1143634 (IL1B gene; T3953C). This suggests a connection between pro-inflammatory factors, specifically polymorphisms in genes initiating the IL1 pathway, and the development of this disorder (Xu, He, 2010; Kapelski et al., 2016).

IL-1RA, acting as an antagonist to the IL-1 receptor, exhibits anti-inflammatory properties. In turn, IL-1α and IL-1β, by binding to the IL-1 receptor, initiate the IL-1 signaling pathway, participating in the implementation of the inflammatory response. Elevated synthesis of IL-1RA blocks this pathway, leading to inhibition of the immune response and weakening of the inflammatory process.

In the analysis of the interaction of the studied proteins, no differences in energy outputs were observed between standard IL-1RA and IL-1RA with rs315952 interacting with IL-1R1. When standard IL-1RA interacts with the IL-1R1+IL-1RAP complex, a lower energy value is observed compared to the case with the polymorphism, presumably indicating a weakening of the interaction between IL-1RA and IL-1R1+IL-1RAP. Notably, IL-1RA does not interact separately with IL-1RAP.

IL-1RA protein, upon binding to IL-1R1 and IL-1R1+IL- 1RAP, inhibits the binding of IL-1 and, consequently, the activation of the IL-1 signaling pathway (Weber et al., 2010). In schizophrenia, the appearance of a single nucleotide polymorphism in the IL-1RA gene (p.Ser130Arg) may lead to the formation of a weakened complex between IL-1RA and associated receptors IL-1R1+IL-1RAP. This, presumably, could subsequently trigger the IL-1 signaling pathway and, as a result, the development of an uncontrolled immune response.

The results of the study showed that the functions of interleukin- 1, namely the interactions of IL-1 family proteins, may be associated with structural changes in the corresponding genes. The analysis of SNP associations of these genes with schizophrenia, together with information about the influence of inflammation on the mechanisms of its development, can serve as a theoretical basis for a more detailed and careful study of the mechanisms of the inflammatory response.

## Conclusion

It is known that in silico mutagenesis and the comparison of changes in interaction energies between the standard and mutated variants shed light on the mechanisms underlying the development of several diseases. The results obtained in this study demonstrate that in schizophrenia, structural changes in genes may influence the functions of interleukin-1 (protein interactions within the IL-1 family). This, in turn, allows correlating existing data on the impact of inflammation on the development of schizophrenia with associations of SNPs in genes related to the IL-1 family. The conducted research makes a theoretical contribution to the understanding of the details of the mechanisms involved in the inflammatory response in schizophrenia, and the results may serve as a basis for further studies (both in silico and experimental) in this field.

## Conflict of interest

The authors declare no conflict of interest.

## References

Acuner Ozbabacan S.E., Gursoy A., Nussinov R., Keskin O. The structural
pathway of interleukin 1 (IL-1) initiated signaling reveals
mechanisms of oncogenic mutations and SNPs in inflammation and
cancer. PLoS Comput. Biol. 2014;10(2):e1003470. DOI 10.1371/
journal.pcbi.1003470

Allan S.M., Tyrrell P.J., Rothwell N.J. Interleukin-1 and neuronal injury.
Nat. Rev. Immunol. 2005;5(8):629-640. DOI 10.1038/nri1664

Aytuna A.S., Gursoy A., Keskin O. Prediction of protein-protein interactions
by combining structure and sequence conservation in protein
interfaces. Bioinformatics. 2005;21(12):2850-2855. DOI 10.1093/
bioinformatics/bti443

Baspinar A., Cukuroglu E., Nussinov R., Keskin O., Gursoy A. PRISM:
a web server and repository for prediction of protein-protein interactions
and modeling their 3D complexes. Nucleic Acids Res. 2014;
42(W1):W285-W289. DOI 10.1093/nar/gku397

Ben Nejma M., Zaabar I., Zaafrane F., Thabet S., Mechri A., Gaha L.,
Ben Salem K., Bel Hadj Jrad B. A gender-specific association of
interleukin 1 receptor antagonist polymorphism with schizophrenia
susceptibility. Acta Neuropsychiatr. 2013;25(6):349-355. DOI
10.1017/neu.2012.32

Bochkov N.P. Clinical Genetics. Moscow: GEOTAR-Media, 2011 (in
Russian)

Chu C.S., Li D.J., Chu C.L., Wu C.C., Lu T. Decreased IL-1ra and
NCAM-1/CD56 serum levels in unmedicated patients with schizophrenia
before and after antipsychotic treatment. Psychiatry Investig.
2018;15(7):727-732. DOI 10.30773/pi.2017.11.10

Dinarello C.A. The interleukin-1 family: 10 years of discovery.
FASEB J. 1994;8(15):1314-1325

Dinarello C.A. Overview of the IL-1 family in innate inflammation and
acquired immunity. Immunol. Rev. 2018;281(1):8-27. DOI 10.1111/
imr.12621

Fatjó-Vilas M., Pomarol-Clotet E., Salvador R., Monté G.C., Gomar
J.J., Sarró S., Ortiz-Gil J., Aguirre C., Landín-Romero R.,
Guerrero-Pedraza A., Papiol S., Blanch J., McKenna P.J., Fañanás L.
Effect of the interleukin-1β gene on dorsolateral prefrontal cortex
function in schizophrenia: a genetic neuroimaging study. Biol. Psychiatry.
2012;72(9):758-765. DOI 10.1016/j.biopsych.2012.04.035

Frodl T., Amico F. Is there an association between peripheral immune
markers and structural/functional neuroimaging findings? Prog.
Neuropsychopharmacol. Biol. Psychiatry. 2014;48:295-303. DOI
10.1016/j.pnpbp.2012.12.013

Gao M., Skolnick J. New benchmark metrics for protein-protein docking
methods. Proteins. 2011;79(5):1623-1634. DOI 10.1002/prot.
22987

Hadarovich A.Y., Kalinouski A.A., Tuzikov A.V. Protein homodimers
structure prediction based on deep neural network. Informatika =
Informatics. 2020;17(2):44-53. DOI 10.37661/1816-0301-2020-17-
2-44-53 (in Russian)

Hudson Z.D., Miller B.J. Meta-analysis of cytokine and chemokine
genes in schizophrenia. Clin. Schizophr. Relat. Psychoses. 2018;
12(3):121-129B. DOI 10.3371/CSRP.HUMI.070516

Kapelski P., Skibinska M., Maciukiewicz M., Wilkosc M., Frydecka
D., Groszewska A., Narozna B., Dmitrzak-Weglarz M., Czerski
P., Pawlak J., Rajewska-Rager A., Leszczynska-Rodziewicz A.,
Slopien A., Zaremba D., Twarowska-Hauser J. Association study of
functional polymorphisms in interleukins and interleukin receptors
genes: IL1A, IL1B, IL1RN, IL6, IL6R, IL10, IL10RA and TGFB1 in
schizophrenia in Polish population. Schizophr. Res. 2015;169(1-3):
1-9. DOI 10.1016/j.schres.2015.10.008

Kapelski P., Skibinska M., Maciukiewicz M., Pawlak J., Dmitrzak-Weglarz
M., Szczepankiewicz A., Zaremba D., Twarowska-Hauser J.
An association between functional polymorphisms of the interleukin
1 gene complex and schizophrenia using transmission disequilibrium
test. Arch. Immunol. Ther. Exp. (Warsz.). 2016;64(Suppl.1):
161-168. DOI 10.1007/s00005-016-0434-6

Katila H., Hänninen K., Hurme M. Polymorphisms of the interleukin-1
gene complex in schizophrenia. Mol. Psychiatry. 1999;4(2):179-
181. DOI 10.1038/sj.mp.4000483

Kim S.J., Lee H.J., Koo H.G., Kim J.W., Song J.Y., Kim M.K.,
Shin D.H., Jin S.Y., Hong M.S., Park H.J., Yoon S.H., Park H.K.,
Chung J.H. Impact of IL-1 receptor antagonist gene polymorphism
on schizophrenia and bipolar disorder. Psychiatr Genet. 2004;14(3):
165-167. DOI 10.1097/00041444-200409000-00009

Krieger E., Vriend G. YASARA View – molecular graphics for all devices
– from smartphones to workstations. Bioinformatics. 2014;
30(20):2981-2982. DOI 10.1093/bioinformatics/btu426

Kuzu G., Gursoy A., Nussinov R., Keskin O. Exploiting conformational
ensembles in modeling protein-protein interactions on the proteome
scale. J. Proteome Res. 2013;12(6):2641-2653. DOI 10.1021/
pr400006k

Liu X., Nemeth D.P., McKim D.B., Zhu L., DiSabato D.J., Berdysz O.,
Gorantla G., Oliver B., Witcher K.G., Wang Y., Negray C.E., Vegesna
R.S., Sheridan J.F., Godbout J.P., Robson M.J., Blakely R.D.,
Popovich P.G., Bilbo S.D., Quan N. Cell-type-specific interleukin 1
receptor 1 signaling in the brain regulates distinct neuroimmune activities.
Immunity. 2019;50(2):317-333.e6. DOI 10.1016/j.immuni.
2018.12.012

McClay J.L., Adkins D.E., Aberg K., Bukszár J., Khachane A.N.,
Keefe R.S., Perkins D.O., McEvoy J.P., Stroup T.S., Vann R.E.,
Beardsley P.M., Lieberman J.A., Sullivan P.F., van den Oord E.J.
Genome-wide pharmacogenomic study of neurocognition as an indicator
of antipsychotic treatment response in schizophrenia. Neuropsychopharmacology.
2011;36(3):616-626. DOI 10.1038/npp.
2010.193

Miyaoka T., Wake R., Hashioka S., Hayashida M., Oh-Nishi A.,
Azis I.A., Izuhara M., Tsuchie K., Araki T., Arauchi R., Abdullah
R.A., Horiguchi J. Remission of psychosis in treatment-resistant
schizophrenia following bone marrow transplantation: a case report.
Front. Psychiatry. 2017;8:174. DOI 10.3389/fpsyt.2017.00174

Müller N. COX-2 inhibitors, aspirin, and other potential anti-inflammatory
treatments for psychiatric disorders. Front. Psychiatry. 2019;
10:375. DOI 10.3389/fpsyt.2019.00375

Papiol S., Molina V., Desco M., Rosa A., Reig S., Gispert J.D., Sanz J.,
Palomo T., Fañanás L. Ventricular enlargement in schizophrenia
is associated with a genetic polymorphism at the interleukin-1 receptor
antagonist gene. Neuroimage. 2005;27(4):1002-1006. DOI
10.1016/j.neuroimage.2005.05.035

Papiol S., Molina V., Rosa A., Sanz J., Palomo T., Fañanás L. Effect of
interleukin-1β gene functional polymorphism on dorsolateral prefrontal
cortex activity in schizophrenic patients. Am. J. Med. Genet.
B Neuropsychiatr. Genet. 2007;144B(8):1090-1093. DOI 10.1002/
ajmg.b.30542

Piñero J., Ramírez-Anguita J.M., Saüch-Pitarch J., Ronzano F., Centeno
E., Sanz F., Furlong L.I. The DisGeNET knowledge platform
for disease genomics: 2019 update. Nucleic Acids Res. 2020;48(D1):
D845-D855. DOI 10.1093/nar/gkz1021

Sasayama D., Hori H., Teraishi T., Hattori K., Ota M., Iijima Y., Tatsumi
M., Higuchi T., Amano N., Kunugi H. Possible association between
interleukin-1β gene and schizophrenia in a Japanese population.
Behav. Brain Funct. 2011;7:35. DOI 10.1186/1744-9081-7-35

Sayers E.W., Beck J., Bolton E.E., Bourexis D., Brister J.R., Canese K.,
Comeau D.C., Funk K., Kim S., Klimke W., Marchler-Bauer A.,
Landrum M., Lathrop S., Lu Z., Madden T.L., O’Leary N., Phan L.,
Rangwala S.H., Schneider V.A., Skripchenko Y., Wang J., Ye J.,
Trawick B.W., Pruitt K.D., Sherry S.T. Database resources of the
National Center for Biotechnology Information. Nucleic Acids Res.
2021;49(D1):D10-D17. DOI 10.1093/nar/gkaa892

Sherry S.T., Ward M.H., Kholodov M., Baker J., Phan L., Smigielski
E.M., Sirotkin K. dbSNP: the NCBI database of genetic variation.
Nucleic Acids Res. 2001;29(1):308-311. DOI 10.1093/nar/
29.1.308

Shirts B.H., Wood J., Yolken R.H., Nimgaonkar V.L. Association study
of IL10, IL1β, and IL1RN and schizophrenia using tag SNPs from
a comprehensive database: suggestive association with rs16944 at
IL1β. Schizophr. Res. 2006;88(1-3):235-244. DOI 10.1016/j.schres.
2006.06.037

Sommer I.E., van Bekkum D.W., Klein H., Yolken R., de Witte L., Talamo
G. Severe chronic psychosis after allogeneic SCT from a schizophrenic
sibling. Bone Marrow Transplant. 2015;50(1):153-154. DOI
10.1038/bmt.2014.221

Tuncbag N., Gursoy A., Nussinov R., Keskin O. Predicting proteinprotein
interactions on a proteome scale by matching evolutionary
and structural similarities at interfaces using PRISM. Nat. Protoc.
2011;6(9):1341-1354. DOI 10.1038/nprot.2011.367

Tuncbag N., Keskin O., Nussinov R., Gursoy A. Fast and accurate modeling
of protein-protein interactions by combining template-interface
docking with flexible refinement. Squirrels. 2012;80(4):1239-1249.
DOI 10.1002/prot.24022

Vilalta A., Brown G.C. Neurophagy, the phagocytosis of live neurons
and synapses by glia, contributes to brain development and disease.
FEBS J. 2018;285(19):3566-3575. DOI 10.1111/febs.14323

Waterhouse A., Bertoni M., Bienert S., Studer G., Tauriello G., Gumienny
R., Heer F.T., Beer T.A.P., Rempfer C., Bordoli L., Lepore R.,
Schwede T. SWISS-MODEL: homology modelling of protein structures
and complexes. Nucleic Acids Res. 2018;46(W1):W296-W303.
DOI 10.1093/nar/gky427

Weber A., Wasiliew P., Kracht M. Interleukin-1 (IL-1) pathway.
Sci. Signal. 2010;3(105):cm1. DOI 10.1126/scisignal.3105cm1

Xu M., He L. Convergent evidence shows a positive association of interleukin-
1 gene complex locus with susceptibility to schizophrenia
in the Caucasian population. Schizophr. Res. 2010;120(1-3):131-
142. DOI 10.1016/j.schres.2010.02.1031

Yoshida M., Shiroiwa K., Mouri K., Ishiguro H., Supriyanto I., Ratta-
Apha W., Eguchi N., Okazaki S., Sasada T., Fukutake M., Hashimoto
T., Inada T., Arinami T., Shirakawa O., Hishimoto A. Haplotypes
in the expression quantitative trait locus of interleukin-1β gene are
associated with schizophrenia. Schizophr. Res. 2012;140(1-3):185-
191. DOI 10.1016/j.schres.2012.06.031

Zanardini R., Bocchio-Chiavetto L., Scassellati C., Bonvicini C.,
Tura G.B., Rossi G., Perez J., Gennarelli M. Association between
IL-1β-511C/T and IL-1RA (86bp)n repeats polymorphisms and
schizophrenia. J. Psychiatr. Res. 2003;37(6):457-462. DOI 10.1016/
s0022-3956(03)00072-4

Zhou Y., Peng W., Wang J., Zhou W., Zhou Y., Ying B. Plasma levels of
IL-1Ra are associated with schizophrenia. Psychiatry. Clin. Neurosci.
2019;73(3):109-115. DOI 10.1111/pcn.12794

